# Small angle X-ray scattering analysis of ligand-bound forms of tetrameric apolipoprotein-D

**DOI:** 10.1042/BSR20201423

**Published:** 2021-01-05

**Authors:** Claudia S. Kielkopf, Andrew E. Whitten, Brett Garner, Simon H.J. Brown

**Affiliations:** 1 Illawarra Health and Medical Research Institute, University of Wollongong, Wollongong, NSW, Australia; 2 School of Chemistry and Molecular Bioscience, University of Wollongong, Wollongong, NSW, Australia; 3 Molecular Horizons, University of Wollongong, Wollongong, NSW, Australia; 4 Australian Nuclear Science and Technology Organisation, Lucas Heights, NSW, Australia

**Keywords:** Apolipoprotein-D, Ligand binding, lipids, Lipocalin, oligomerization, small-angle scattering

## Abstract

Human apolipoprotein-D (apoD) is a glycosylated lipocalin that plays a protective role in Alzheimer’s disease due to its antioxidant function. Native apoD from human body fluids forms oligomers, predominantly a stable tetramer. As a lipocalin, apoD binds and transports small hydrophobic molecules such as progesterone, palmitic acid and sphingomyelin. Oligomerisation is a common trait in the lipocalin family and is affected by ligand binding in other lipocalins. The crystal structure of monomeric apoD shows no major changes upon progesterone binding. Here, we used small-angle X-ray scattering (SAXS) to investigate the influence of ligand binding and oxidation on apoD oligomerisation and conformation. As a solution-based technique, SAXS is well suited to detect changes in oligomeric state and conformation in response to ligand binding. Our results show no change in oligomeric state of apoD and no major conformational changes or subunit rearrangements in response to binding of ligands or protein oxidation. This highlights the highly stable structure of the native apoD tetramer under various physiologically relevant experimental conditions.

## Introduction

**Figure 1:**
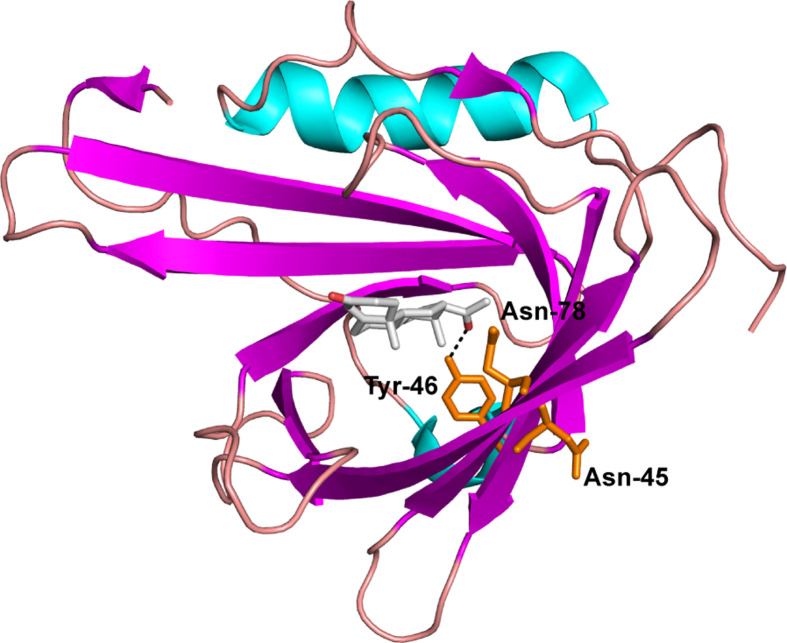
ApoD crystal structure and conformational changes upon progesterone binding ApoD adopts a typical lipocalin fold with an eight-stranded β-barrel and an adjacent α-helix. Three amino acids, Asn-45, Tyr-46 and Asn-78, show a conformational change upon progesterone binding.

Apolipoprotein-D (apoD) is a ∼25 kDa glycoprotein belonging to the protein family of lipocalins [1,2], a family which is characterised by high structural homology and the ability to bind and transport small hydrophobic ligands [3]. ApoD adopts a typical lipocalin fold of a β-barrel ligand pocket and an adjacent α-helix (Figure 1, PDB ID: 2HZQ). Two disulphide bonds connect the N- and C-terminal segments to the β-barrel while a fifth free cysteine forms an intermolecular disulphide bond to apoA-II in plasma [4]. The crystal structure of apoD shows that upon progesterone binding, three side chains change conformation (Figure 1) [1]. ApoD is furthermore consistently glycosylated at two amino acids, with the exact glyco-composition being heterogeneous [5]. ApoD has an antioxidant function and plays a protective role in Alzheimer’s disease [6,7]. Using small angle X-ray scattering (SAXS) and other techniques, we have previously shown that the apo form of native human apoD isolated from breast cyst fluid (BCF) forms a tetrameric oligomer that is stable upon dilution [8]. Oligomerisation is common in the lipocalin family and has been shown in other lipocalins to be influenced by ligand binding, salt concentration and pH [3,9–11]. Specifically, ligand binding affects oligomerisation and vice versa in lipocalins [11,12]. Ligand binding of β-lactoglobulin leads to dimer dissociation [11], and ligand binding of crustacyanin, a pigmentation protein, is critically dependent on dimer formation [12].

Ligand binding may affect not only oligomerisation but also the 3D structure of apoD, even though substantial changes were not observed in the crystal structure of recombinant monomeric apoD upon progesterone binding [1]. Hydrogen–deuterium exchange coupled with mass spectrometry (HDX-MS) has recently shown reduced deuterium exchange in the apoD ligand binding pocket as well as in peripheral regions upon progesterone binding, suggesting a stabilisation of these areas induced by progesterone [13]. Given the reported difficulties in crystallising native apoD [14], X-ray crystallography cannot be readily employed to assess the influence of ligand binding to tetrameric apoD. SAXS, in contrast, is a solution based scattering technique that can determine the size and shape of biomolecules and allows structural modelling. In combination with crosslinking-mass spectrometry and the described model of glycosylated monomeric apoD [15], SAXS provided an opportunity to structurally model the apoD tetramer [8]. However, the impact that ligand binding may have on apoD oligomerisation and overall structure was not investigated, nor was the influence of oxidation of Met-93. Oxidation of Met-93 *in vitro* leads to apoD dimer formation and these dimers are also observed in the brain of Alzheimer's disease patients [16], a function unique to apoD in the lipocalin family. In the present study, we use SAXS to determine if ligand binding or oxidation affects oligomerisation or conformation of native human apoD.

## Materials and methods

The methods for apoD purification and SAXS analysis have been previously published in detail [8]. In the present study, we used the same experimental instrument conditions as described previously [8], in order to allow a direct comparison of the previously published data pertaining to the apo form of apoD with the data presented herein pertaining to the ligand-bound forms of apoD. The pertinent methodological information is provided below in brief.

### Sample preparation

ApoD was purified from human BCF using ion exchange chromatography and size exclusion chromatography (SEC) in SAXS-buffer (50 mM sodium phosphate, 150 mM NaCl, 3% (v/v) glycerol, pH 7.4) and SEC fractions were pooled to a concentration of 1.26 mg/ml (43 µM). A Coomassie stained SDS-PAGE gel of the pooled fractions is shown in Supplementary Figure S1. For each ligand, 1 ml of apoD was diluted ten-fold with SAXS-buffer to a concentration of 4.3 µM. Biliverdin (*K*_D_ unknown [17]), palmitic acid (*K*_D_ 3.3 μM [18]), progesterone (*K*_D_ 1.7 μM [19]) and palmitoyl sphingomyelin (*K*_D_ 1.3 μM [18]) were dissolved in dimethylformamide and added at 10× molar excess (30 µl, final concentration 43 µM) to apoD (10 ml, 4.3 µM). The samples were inverted immediately after adding ligands to prevent ligand precipitation and incubated for 1 h at 22°C while gently inverting. Ligand occupancy was calculated according to (eqn 1):

(1) $$Y=\frac{\left[ApoD\cdot L\right]}{{\left[ApoD\right]}_{total}}=\;\frac{({\left[L\right]}_{total}+{\left[ApoD\right]}_{tot}+K_{d})-\sqrt{{\left({\left[L\right]}_{tot}+{\left[ApoD\right]}_{tot}+K_{d}\right)}^{2}-4\cdot {\left[ApoD\right]}_{tot}\;\cdot {\left[L\right]}_{tot}}}{2\cdot {\left[ApoD\right]}_{tot}}$$

For oxidation, 1 ml of apoD (43 µM) was incubated with H_2_O_2_ (final concentration 100 mM) overnight at 4°C. H_2_O_2_ is known to oxidise free cysteines, lysine, histidines and glycines in addition to methionines [20].

Ligand-bound samples were buffer exchanged to remove unbound ligands and spin-concentrated using Amicon Ultra concentrators with an Ultracel-10 membrane (10 kDa molecular weight cut-off). Oxidised apoD was directly spin-concentrated and all samples were frozen at −80°C. Final protein concentrations of all samples are listed in Table 1. All protein concentrations were measuring using a Pierce BCA assay (ThermoFisher) with bovine serum albumin serial dilution as standard curve according to the manufacturer’s instructions.

**Table 1:** Sample and structural parameters for apoD in the ligand-bound and oxidised forms

	Biliverdin	Palmitic acid	Progesterone	Sphingomyelin	Oxidised (H_2_O_2_)
Loading concentration (mg/ml)	8.44	8.89	8.435	5.01	9.40
Guinier analysis					
*I*(*0*) (cm^−1^)	0.07701 ± 0.00008	0.04668 ± 0.00008	0.04536 ± 0.00009	0.07839 ± 0.0001	0.07375 ± 0.00009
*R*_g_ (Å)	33.4 ± 0.1	33.4 ± 0.2	33.4 ± 0.3	33.3 ± 0.2	33.4 ± 0.1
*q*_min_ (Å^-1^)	0.0099	0.0099	0.0111	0.0123	0.0099
*qR*_g_ max	1.27	1.27	1.27	1.27	1.27
Coefficient of correlation, *R*^2^	0.9997	0.9992	0.9987	0.9994	0.9996
*P*(*r*) analysis					
*I*(*0*) (cm^−1^)	0.07724 ± 0.00008	0.04674 ± 0.00007	0.04213 ± 0.00007	0.07855 ± 0.0001	0.07384 ± 0.00007
*R*_g_ (Å)	33.40 ± 0.05	33.48 ± 0.07	33.24 ± 0.06	33.37 ± 0.05	33.41 ± 0.04
*D*_max_ (Å)	105	106	100	99	103
*q* range (Å^-1^)	0.0099–0.239	0.0099–0.239	0.0099–0.239	0.0135–0.239	0.0099–0.239
*χ*^2^ (total estimate from GNOM)	1.08	1.02	1.14	0.97	1.21
Porod volume estimate (Å^3^)	168000	163000	161000	171000	164000
Molecular weight (kDa), calculated from *I*(*0*) and Abs_280_	86	87	96	103	92

### Small angle X-ray scattering data collection and analysis

SEC-SAXS data in coflow mode [21] were collected at the SAXS/WAXS beamline at the Australian Synchrotron, Clayton, Australia [22]. Samples were thawed on ice and spun for 10 min at 16 k × *g.* A GE Superdex 200 5/150 column was equilibrated to SAXS-buffer and 100 µl of ligand bound/oxidised apoD were applied to the column. SAXS parameters are listed in Supplementary Table S1. Primary data reduction was done in ScatterBrain (2.710), all other data analyses were performed using ATSAS package 2.8.2 [23]. For buffer subtraction, 30 frames before protein elution were selected, averaged and subtracted from the averaged data. Guinier and Porod distance distribution analyses were carried out using Primusqt. The molecular weights were calculated using *I*(*0*) [24], using contrasts and partial specific volumes calculated using MULCh [25]. All scattering data and parameters were deposited to SASBDB under the accession numbers SASDHJ5 (biliverdin), SASDHK5 (oxidised apoD), SASDHL5 (palmitic acid), SASDHM5 (progesterone) and SASDHN5 (sphingomyelin).

### Graphs and image analysis

Graphs were created using GraphPad Prism. Errors are based on counting statistics and error bars are not shown if they are smaller than symbol sizes.

## Results

**Figure 2:**
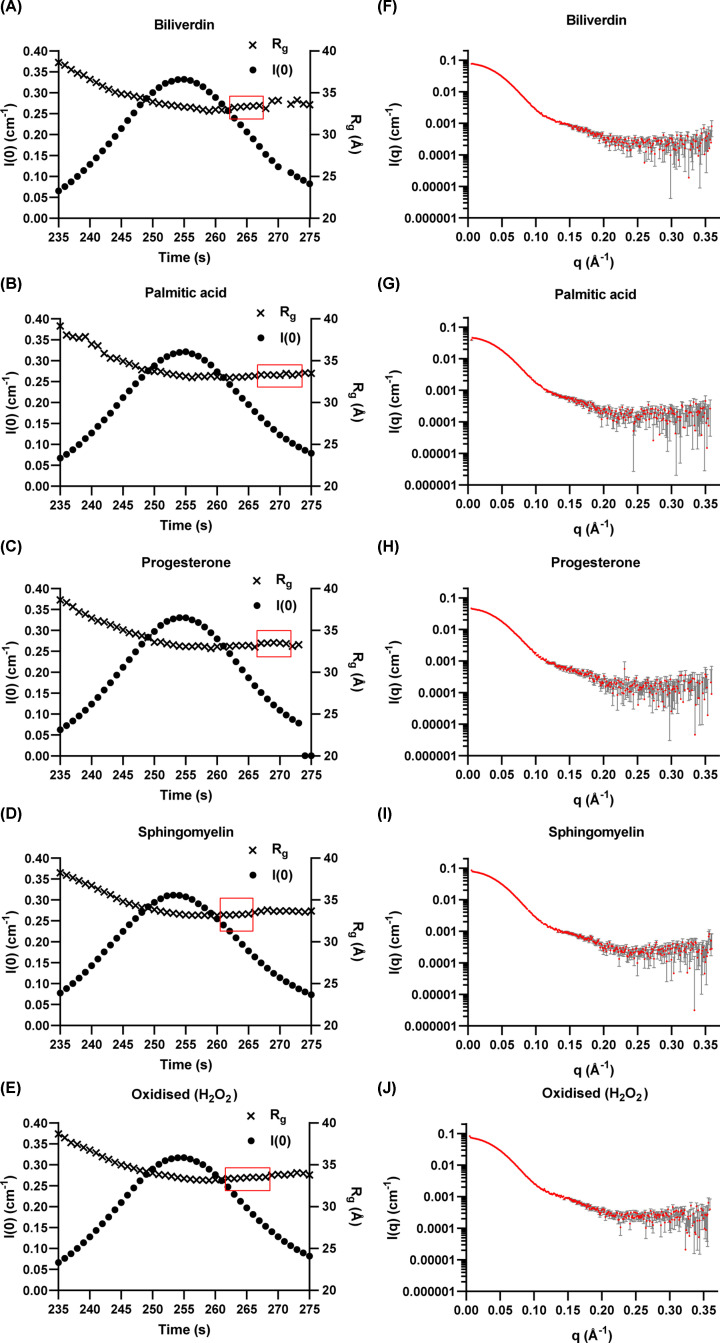
Experimental SAXS data for ligand-bound and oxidised apoD tetramer (**A**–**E**) Scattering intensity *I*(*0*) (dots) and radius of gyration *R*_g_ (crosses) over apoD elution on an S200 5/150 SEC column for biliverdin-, palmitic acid-, progesterone-, sphingomyelin-bound and oxidised apoD. Areas with constant *R*_g_ that showed no inter-particle repulsion were selected for averaging (marked in red). (**F–J**) Total experimental scattering profile of ligand-bound or oxidised apoD. All scattering curves show the same overall shape and extent. Error bars in scattering profiles are based on counting statistics and error bars are not shown if they are smaller than symbol sizes.

To evaluate if ligand binding or oxidation influences apoD oligomeric state or causes structural rearrangement of apoD subunits, apoD was incubated with ligands (biliverdin, palmitic acid, progesterone or sphingomyelin) or oxidised using H_2_O_2_, and subjected to SEC-SAXS analysis. All SAXS parameters are presented in Supplementary Table S1 and *R*_g_ and scattering intensity *I*(*0*) across the SEC peak for all samples are shown in Figure 2A–E. No additional peaks other than the main peak at 255 s were observed (data not shown). To select appropriate areas for averaging, regions with constant *R*_g_ were identified. These areas were further assessed for inter-particle repulsion using Guinier and Porod analyses after averaging. Areas to be averaged were selected where no signs of aggregation and inter-particle repulsion were present. Final averaged frames for further analysis are marked in red (Figure 2A–E), and total scattering profiles for these averaged frames are shown in Figure 2F–J.

**Figure 3:**
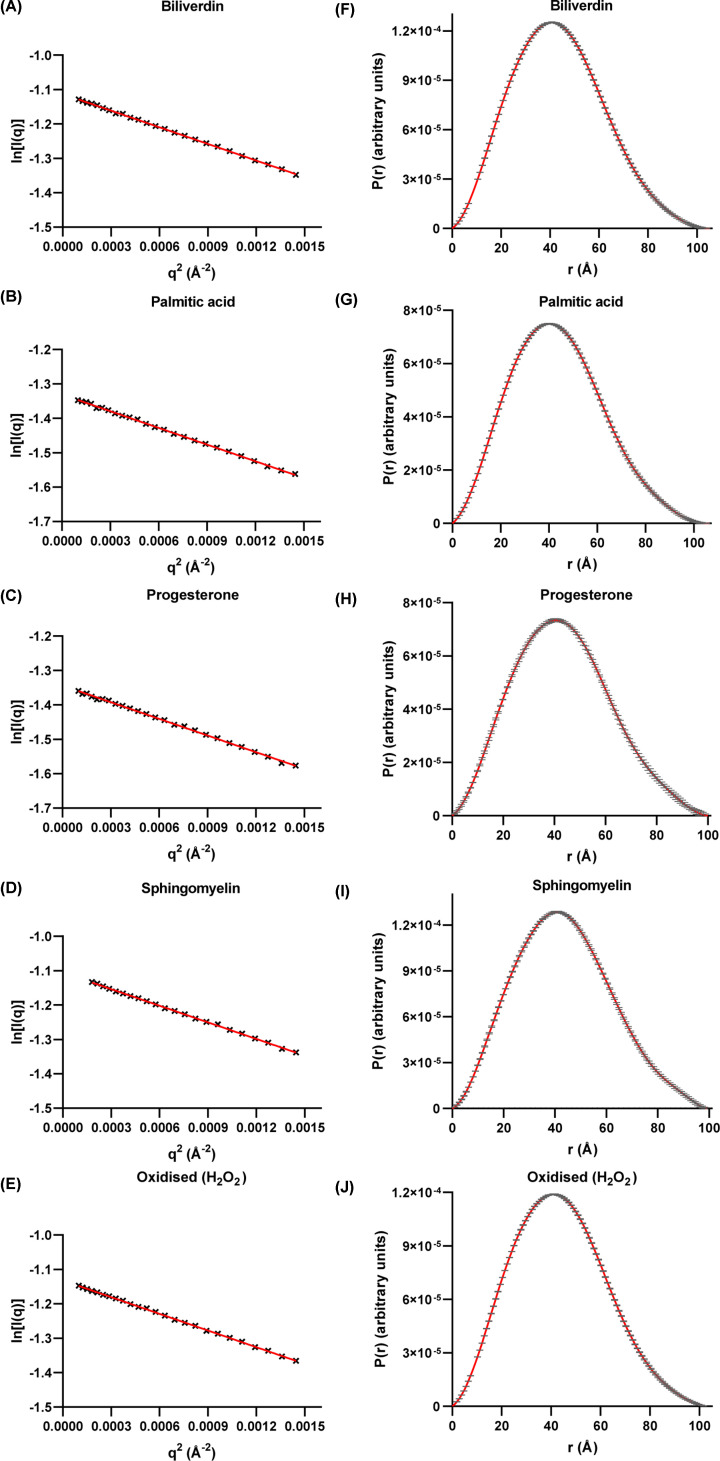
Guinier and Porod analysis for ligand-bound and oxidised apoD (**A**–**E**) Guinier plots of apoD bound to a ligand or oxidised apoD. Linear regression showed no up- or down-turn of the curve, indicating no aggregation or inter-particle repulsion. (**F–J**) *P*(*r*) functions of apoD bound to a ligand or oxidised apoD displayed a symmetric profile indicative of a mainly globular scattering molecule. All curves approach zero smoothly at high *q*, indicating the absence of inter-particle repulsion. Error bars in *P*(*r*) functions are based on counting statistics and error bars are not shown if they are smaller than symbol sizes.

Averaged and buffer-subtracted data for ligand-bound and oxidised apoD were evaluated in the Guinier region at small *q* values where no indication of aggregation or inter-particle repulsion was observed (Figure 3A–E). *P*(*r*) functions for each sample showed a bell shape that smoothly approached zero at *D*_max_ (Figure 3F–J). These curves correspond to a globular molecule and showed no signs of inter-particle repulsion.

**Figure 4:**
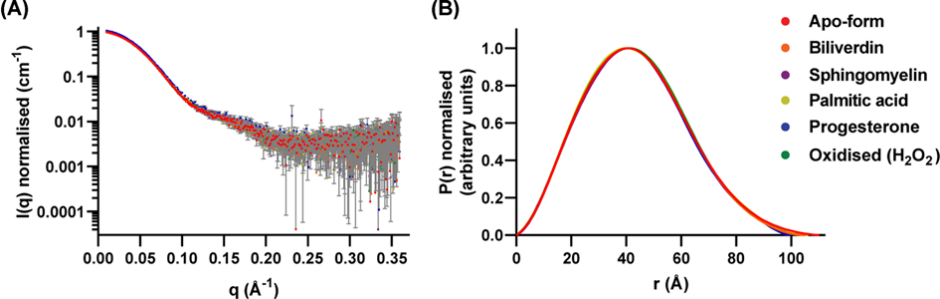
Overlay of normalised scattering data and *P*(*r*) curves (**A**) All scattering data for apoD in the apo-, ligand-bound and oxidised form were normalised to *I*(*0*), as calculated by Porod analysis, and overlaid. The shape of the scattering profiles was identical. (**B**) *P*(*r*) functions for apoD in the apo, ligand-bound and oxidised form were normalised to *P*(*r*)_max_ and overlaid. The shape of the Porod distribution was identical. However, there were slight variances at high radii. Error bars in scattering profiles are based on counting statistics and error bars are not shown if they are smaller than symbol sizes. Data for the apo-form of apoD are derived from Kielkopf et al. [8]

Scattering profiles and *P*(*r*) curves for ligand-free apoD and ligand-bound and oxidised apoD were normalised to *I*(*0*) (as determined by *P*(*r*) analysis) and *P*(*r*)_max_, respectively. Thereby, scattering profiles and *P*(*r*) curves of ligand-bound or oxidised apoD can be compared with ligand-free apoD more easily (Figure 4). Neither ligand binding nor oxidation using H_2_O_2_ changed the scattering profile substantially (Figure 4A). A small difference can be noted in progesterone-bound apoD. The *P*(*r*) curves remained largely the same upon addition of ligand and oxidation (Figure 4B). Slight differences were only observed at high values of *r*, possibly indicating a reduction in *D*_max_ of ligand-bound and oxidised apoD compared with ligand-free apoD.

*R*_g_ values calculated from *P*(*r*) analysis, *D*_max_ and estimated Porod volumes of all studied samples are listed in Table 2. There were small changes in *R*_g_, *D*_max_ and Porod volume in ligand-bound or oxidised apoD compared with ligand-free apoD. Ligand-bound or oxidised apoD showed a decrease in *R*_g_ of maximally 0.47 Å (progesterone-bound apoD). *D*_max_ was decreased in all ligand-bound or oxidised samples of maximally 11 Å (sphingomyelin-bound apoD). The Porod volume varied from a decrease of 8000 Å^3^ (progesterone-bound apoD) to an increase of 2000 Å^3^ (sphingomyelin-bound apoD) compared with ligand-free apoD. Due to the inherent uncertainties of *R*_g_, *D*_max_ and Porod volume [26–31], none of these changes are considered significant.

**Table 2:** Comparison of SAXS parameters of ligand-free, ligand-bound and oxidised apoD

Sample	*R*_g_ (Å)	*D*_max_ (Å)	Porod volume estimate (Å^3^)
^1^Apo-form	33.71 ± 0.07	110	169,000
Biliverdin	33.40 ± 0.05	105	168,000
Palmitic acid	33.48 ± 0.07	106	163,000
Progesterone	33.24 ± 0.06	100	161,000
Sphingomyelin	33.37 ± 0.05	99	171,000
Oxidised (H_2_O_2_)	33.41 ± 0.04	103	164,000

1 Data derived from Kielkopf et al. [8]

Parameters are derived from *P*(*r*) analysis.

## Discussion

SAXS as a solution-based scattering technique is a powerful method to determine size and shape of proteins and protein complexes. Our previously published SAXS experiments of ligand-free apoD revealed the nature of apoD as a tetrameric oligomer with an *R*_g_ of ∼33 Å and a globular structure [8]. The molecular weight of apoD calculated from the Porod analysis and *I*(*0*) was consistently determined at ∼97 kDa, which is in good agreement with data derived from other biophysical analyses [8].

In the present study, when apoD was incubated with one of the apoD ligands biliverdin, palmitic acid, progesterone or sphingomyelin, or oxidised using H_2_O_2_, no significant changes in the scattering profile, Porod curve, *R*_g_ or *D*_max_ were detected (Figure 4 and Table 2). No additional peaks were observed in the size exclusion chromatogram (data not shown). These results indicate that ligand binding and oxidation do not lead to a change in apoD oligomeric state and do not cause substantial conformational changes or subunit rearrangements. Interestingly, HDX-MS showed a stabilisation of apoD dynamics upon progesterone binding but no major conformational changes [13], which is in agreement with the SAXS data presented here.

One potential confounding factor in the SAXS experiment could be an endogenous ligand co-purified with apoD from BCF. In the past, we characterised hydrophobic extracts from apoD using untargeted liquid chromatography-tandem mass spectrometry analysis. Progesterone or other potential apoD ligand such as free fatty acids or phospholipids were not identified (data not shown).

While the ligand occupancy of apoD during incubation with the ligands was calculated to be between 92% and 96%, the occupancy during the experiment may be lower due to the need to remove ligand and solvent before the SEC-SAXS experiment. HDX-MS experiments show that progesterone binding persists over a 2-h experiment at final concentrations of 10.7 μM (apoD) and 3 μM (progesterone) [13]. Notably, organic solvent was present during *K*_D_ measurements for most apoD ligands, thereby influencing the off-rate and the *K*_D_ [32–34]. In the here presented SAXS experiments, the organic solvent was removed in the process, thereby reducing the solubility of the hydrophobic ligand in the aqueous surrounding and limiting the ligand off rate. Including free ligand in the running buffer is not practical for hydrophobic ligands as the amount of solvent required could influence the structural integrity of the protein [35–37].

SAXS studies of the lipocalins lipocalin-type prostaglandin D synthase (PGDS), bovine β-lactoglobulin A and retinol-binding protein have been performed upon binding of retinoic acid. In addition, SAXS was performed on PGDS upon binding of bilirubin and biliverdin [38]. SAXS showed an *R*_g_ reduction in PGDS of up to 2.1 Å upon ligand binding, but no changes were observed in β-lactoglobulin and retinol-binding protein. Further SAXS experiments combined with NMR using PGDS identified tightening in loop and helix regions of PGDS when bound to biliverdin [39]. Taking the inherent uncertainty of *R*_g_ and *D*_max_ into account, our study of apoD showed no difference in *R*_g_ and *D*_max_, similarly to β-lactoglobulin and retinol-binding protein. Although the exact ligand occupancy of apoD was not quantitatively assessed in the present work, previous studies indicate that hydrophobic ligands such as those used by us herein are much less soluble in aqueous environment, consistent with their stable binding within the lipophilic binding pocket of apoD; a feature that is common among other members of the lipocalin family [38,39]. Similar to apoD, X-ray crystallography of PGDS showed conformational changes in single amino acids and in a single loop when PGDS was bound to a substrate analogue [40] or fatty acids (pdb accession number 3O22). These data underline the importance of SAXS as a sensitive solution-based structural technique in characterising interactions of proteins with hydrophobic ligands. Furthermore, these examples suggest that the structural response of a lipocalin to ligand binding depends on the specific lipocalin and ligand, as is the case for other proteins [41]. This may reflect the fact that many lipocalins bind a spectrum of ligands, potentially resulting in a variety of scenarios upon ligand binding.

In conclusion, ligand binding or oxidation did not induce significant changes to the native oligomeric status of apoD, nor did these parameters induce substantial conformational change or subunit rearrangements as determined by SAXS analysis. The present study highlights the highly stable structure of the native apoD tetramer under various physiologically relevant experimental conditions.

## Supplementary Material

Supplementary Figure S1

## Data Availability

All scattering data and parameters were deposited to SASBDB under the accession numbers SASDHJ5 (biliverdin), SASDHK5 (oxidised apoD), SASDHL5 (palmitic acid), SASDHM5 (progesterone) and SASDHN5 (sphingomyelin). All other data required for replicating this study are given in the manuscript.

## Abbreviations

apoD: apolipoprotein-D
BCF: breast cyst fluid
HDX-MS: hydrogen–deuterium exchange coupled with mass spectrometry
PGDS: prostaglandin D synthase
SAXS: small-angle X-ray scattering
SEC: size-exclusion chromatography
