# Supplementary material for: Small angle X-ray scattering analysis of ligand-bound forms of tetrameric apolipoprotein-D
Source: Biosci Rep. 2021 Jan 5;41(1):BSR20201423. doi: 10.1042/BSR20201423 (PMC7786332; doi:10.1042/BSR20201423)
Supplement: Supplementary Figure S1 [file BSR-2020-1423_supp.pdf]

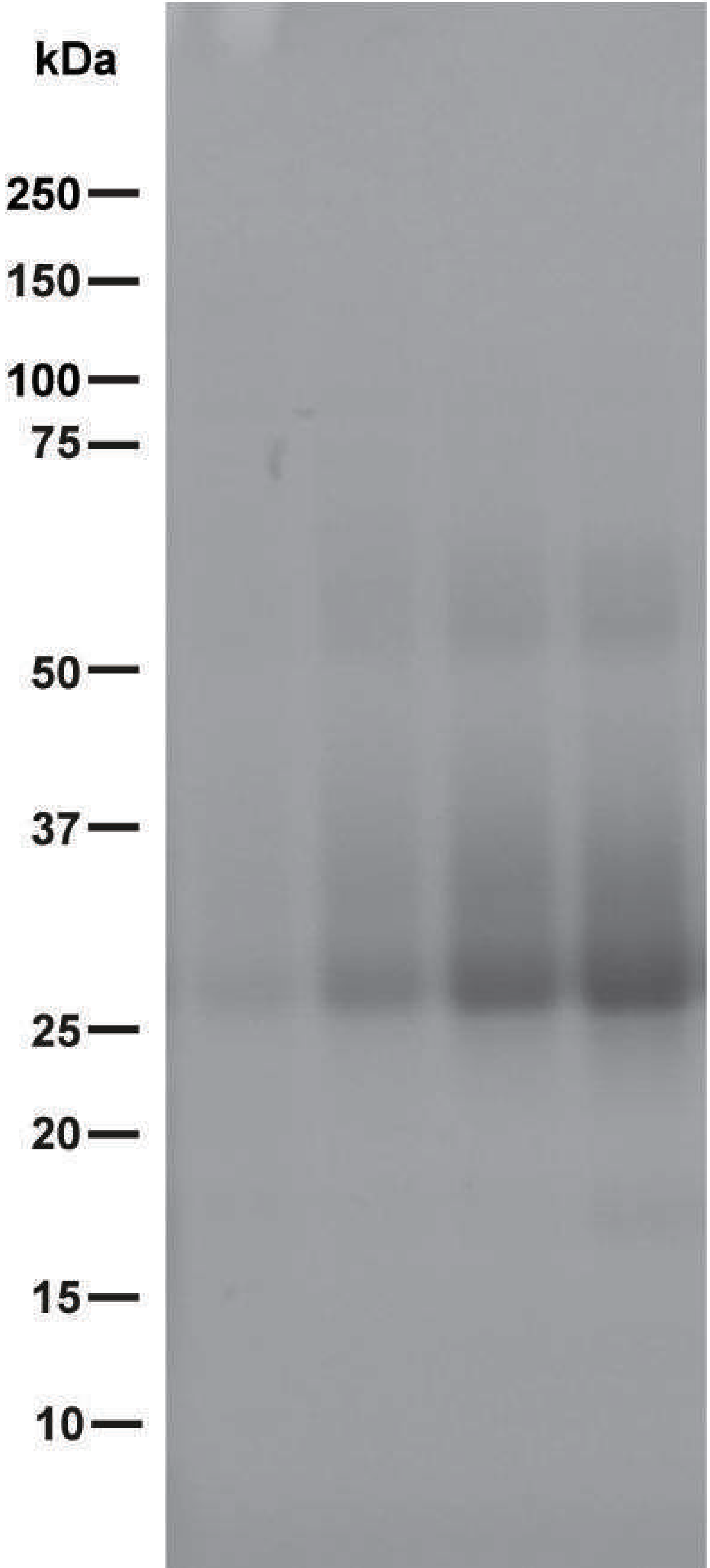
kDa
250
150
100
75
50
37
25
20
15
10

Figure S1. SDS-PAGE stained with Coomassie of the SEC fractions to be pooled for SAXS analysis. ApoD was purified using IEX and SEC and eluted fractions from SEC were analysed on SDS-PAGE stained with Coomassie staining. Fractions to be pooled are shown here. ApoD bands show consistent glycosylation with band smearing likely representing glyco-microheterogeneity.
